# Identifying Suitable Patients for Overcoming Androgen Deprivation Monotherapy in De Novo Metastatic Hormone-Sensitive Prostate Cancer

**DOI:** 10.3390/jpm14050517

**Published:** 2024-05-13

**Authors:** Donghyun Lee, Bumjin Lim, Tuan Thanh Nguyen, Se Young Choi

**Affiliations:** 1 Department of Urology, Hallym University Kangnam Sacred Heart Hospital, Hallym University College of Medicine, Seoul 07441, Republic of Korea; leedh@hallym.or.kr; 2 Department of Urology, Asan Medical Center, University of Ulsan College of Medicine, Seoul 05505, Republic of Korea; lbj1986@amc.seoul.kr; 3 Department of Urology, Cho Ray Hospital, University of Medicine and Pharmacy, Ho Chi Minh City 700000, Vietnam; nguyen.thanh.tuan@ump.edu.vn; 4 Department of Urology, Chung-Ang University Hospital, Chung-Ang University College of Medicine, Seoul 06973, Republic of Korea

**Keywords:** androgen deprivation therapy, castration-resistant prostate cancer, LHRH agonist, life expectancy, overall survival

## Abstract

Background: Although metastatic hormone-sensitive prostate cancer (mHSPC) treatments have evolved, androgen deprivation therapy (ADT) remains a widely used regimen. Therefore, this study sought patients who did not progress to castration-resistant prostate cancer (CRPC) but received ADT monotherapy and factors affecting overall survival (OS) in de novo mHSPC. Methods: De novo mHSPC patients who received ADT treatment were included. ADT included luteinizing hormone-releasing hormone agonists with or without anti-androgen. The total cohort was divided into two groups relative to CRPC progression within two years. Logistic analysis was used to identify factors that did not progress CRPC within two years. Cox regression was used to assess the independent predictors for OS. Results: The total cohort was divided into the no-CRPC within two years group (*n* = 135) and the CRPC within two years group (*n* = 126). Through multivariate logistic analysis, the life expectancy (odds ratio [OR] 0.95, 95% CI 0.91–0.99, *p* = 0.014) and Gleason scores (≥9 vs. ≤8; OR 0.43, 95% CI 0.24–0.75, *p* = 0.003) were associated with the group without castration-resistant prostate cancer progression within two years. The multivariate Cox model revealed that life expectancy (hazard ratio [HR] 0.951, 95% CI 0.904–0.999, *p* = 0.0491), BMI (HR 0.870, 95% CI 0.783–0.967, *p* = 0.0101), and CCI (≥2 vs. <2; HR 2.018, 95% CI 1.103–3.693, *p* = 0.0227) were significant predictive factors for OS. Conclusions: Patients with long life expectancy and a Gleason score of 9 or more were more likely to develop mCRPC while alive. Patients with short life expectancy, low BMI, and worsening comorbidity were more likely to die before progressing to CRPC. Although intensified treatment is essential for oncologic outcomes in mHSPC, shared decision making is integral for patients who may not benefit from this treatment.

## 1. Introduction

More than 1.4 million new cases of prostate cancer have been reported globally, and approximately 375,000 related deaths were reported in 2020 [1]. Upon initial diagnosis, de novo metastatic hormone-sensitive prostate cancer (mHSPC) accounts for about 4% in Western countries and 9% in South Korea [2,3]. Among metastatic castration-resistant prostate cancer (mCRPC), approximately 35% of patients were initially diagnosed with localized prostate cancer that progressed into metachronous mHSPC [4]. However, patients with mCPRC after de novo mHSPC accounted for 28% [4]. Clinical outcomes and genomic mutational profiles may differ between patients with de novo mHSPC or metachronous mHSPC [5,6]. As such, the process leading to mHSPC has diverse and heterogeneous aspects.

In recent years, there has been a significant shift in the treatment landscape for metastatic hormone-sensitive prostate cancer (mHSPC). Several pivotal studies have emerged, demonstrating notable survival gains with the introduction of new medications such as abiraterone, enzalutamide, and apalutamide, thereby establishing a new standard of care for mHSPC [7]. Furthermore, there has been increasing attention on triplet treatment approaches involving androgen deprivation therapy (ADT) and androgen receptor-targeted agents (ARTAs) in combination with docetaxel [8]. ARTAs, designed to specifically target the androgen receptor, represent a novel class of treatments for prostate cancer, notable for their oral formulation, which often eliminates the need for hospitalization. This characteristic makes them more appealing to patients compared to traditional intravenous treatments.

Recent advancements in prostate cancer therapy have culminated in the development of triplet therapy, as evidenced by trials such as PEACE-1, investigating abiraterone, and ARASENS, evaluating darolutamide [8]. Triplet therapy entails a combination of ADT with docetaxel, supplemented by the addition of an ARTA. Clinical trials have consistently demonstrated that this approach yields superior overall survival (OS) rates compared to standard-of-care treatments. As a result, treatment guidelines strongly endorse the adoption of intensified therapies, such as doublet or triplet regimens, for patients with mHSPC [9,10].

Despite the growing acceptance of intensified treatment approaches, it is noteworthy that more than fifty percent of patients with mHSPC currently do not receive the standard of care as outlined by established clinical guidelines in the United States [11]. This underscores the importance of ongoing efforts to bridge the gap between evidence-based recommendations and clinical practice, ensuring that all eligible patients have access to optimal treatment strategies.

Currently, ADT monotherapy is the predominant treatment strategy globally for managing mHSPC [12]. Although the demonstrated survival benefit provided by this treatment paradigm should certify this approach as a clear standard of care, there are barriers to intensified treatment in clinical settings. Consequently, a precise classification of mHSPC patients is imperative to identify candidates who may benefit from more aggressive treatment approaches. This study focuses on identifying patients with de novo mHSPC who have not progressed to CRPC following ADT monotherapy, and investigates the factors influencing OS in this cohort.

## 2. Materials and Methods

### 2.1. Ethics Statement

Asan Medical Center’s ethical board approved this study (IRB no. 2023-1590). The need for written consent was waived, and patient data were anonymized before analysis. The research process adhered to the ethics of the Helsinki Declaration.

### 2.2. Patient Enrollment

A retrospective review identified prostate cancer patients who received primary ADT in the Asan Medical Center between 2008 and 2012. Patients who underwent radical prostatectomy or definitive radiation therapy with ADT were excluded. Overall, 261 de novo mHSPC patients treated with ADT were included in this study. ADT only entailed luteinizing hormone-releasing hormone agonists with or without anti-androgen (bicalutamide). Clinical data for age, body mass index, past medical history (including the Charlson Comorbidity Index [CCI]), serum PSA levels, visceral metastasis, and Gleason scores were collected.

Patient follow-ups included serum PSA, computed tomography, or bone scans. CRPC was defined as a rising PSA in two consecutive measurements taken at least one week apart. PSA was required to be ≥2 ng/mL and ≥25% above the nadir value despite castrate testosterone (serum testosterone < 50 ng/dL). The total cohort was divided into two groups relative to CRPC progression within two years. The Kaplan–Meier analysis estimated CRPC probability and overall survival (OS) was calculated from the prostate cancer diagnosis to death. The general population’s life expectancy by age and sex was available through the Korean Statistical Information Service [https://kosis.kr/eng/ (accessed on 20 December 2023)]. Korea’s life table was completed through the following calculations, allowing us to determine life expectancy.

1. Calculation of Age-Specific Mortality Rate (mx): The age-specific mortality rate represents the ratio of deaths (Dx) in each age group to the corresponding population (Px). It reflects the mortality rate for a specific age group.

$$mx=\frac{Dx}{Px}$$

- Dx: Number of deaths at age.
- *Px*: Population at age x.

2. Calculation of Age-Specific Mortality Probability (qx): Age-specific mortality probability is derived from the age-specific mortality rate (mx). It is calculated using the mortality rate to adjust for age distortion.
  $$qx’=\frac{mx}{1+mx/2}$$

- qx’: Adjusted age-specific mortality probability
- mx: Age-specific mortality rate

3. Calculation of Age-Specific Survivor Count (lx): The survivor count represents the number of individuals surviving in each age group. It is calculated by subtracting the number of deaths (dx) from the previous age group’s survivor count.

lx+1=lx−dx

(dx=lx×qx)

4. Calculation of Age-Specific Stationary Population (Lx): The stationary population for ages 100 and above is calculated until *Lx* reaches 0, representing the population that has reached the end of life.

$$Lx=\frac{lx+lx+1}{2}$$

5. Calculation of Total Person years Lived (Tx): Total person years lived represents the sum of stationary populations across all age groups.

$$Tx=\sum_{x}^{\infty }Lx$$

6. Calculation of Life Expectancy (ex): Life expectancy is derived by dividing the total person years lived by the initial survivor count.
  $$ex=\frac{Tx}{lx}$$

### 2.3. Statistical Analysis

Patient and tumor baseline clinicopathological characteristics were expressed as means ± standard deviation or percentages. The normal distribution of data was determined using the Kolmogorov–Smirnov test, and pairs of groups were compared using t-tests or the Mann–Whitney U-test. Groups were compared by chi-squared tests for categorical variables. Logistic regression analysis identified factors that did not progress CRPC within two years. Cox proportional hazard regression analysis assessed the independent predictors regarding OS in the no-CRPC cohort. All statistical analyses were performed with R (version 4.3.1; R Project for Statistical Computing, Vienna, Austria). A *p*-value < 0.05 was considered statistically significant.

## 3. Results

The total cohort was divided into the no-CRPC within two years group (*n* = 135) and the CRPC within two years group (*n* = 126). Patient and tumor characteristics are presented in Table 1. The median follow-up period was 49.4 months, and the median age was 69.2 ± 8.7 years. The no-CRPC group included patients older than the CRPC group (70.5 ± 7.3 vs. 67.8 ± 10.0, *p* = 0.015), but their life expectancies were not significantly different (14.7 ± 5.3 vs. 19.4 ± 29.0, *p* = 0.080). The no-CRPC group had lower rates of Gleason scores of 9 or more than the CRPC group (49.6% vs. 69.8%, *p* = 0.001). The two groups had similar BMI, ECOG-PS, CCI, initial PSA, and visceral metastasis values.

**Figure 1:**
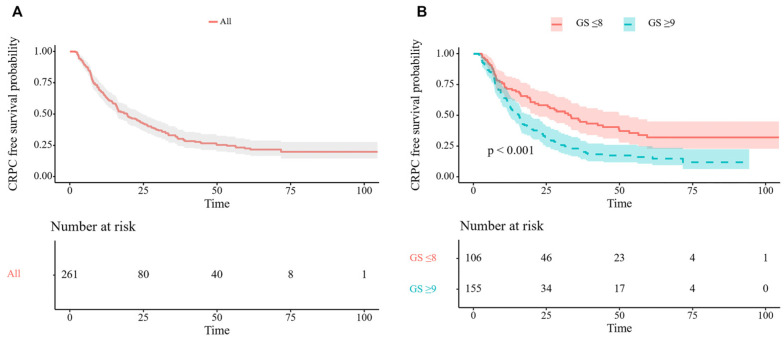
Kaplan–Meier survival curve for castration-resistant prostate cancer-free survival in the total cohort (**A**) and by Gleason score (**B**). CRPC, castration-resistant prostate cancer; GS, Gleason score.

The median survival time until CRPC was 19.5 months (95% confidence interval [CI] 15.0–24.0). The estimated cumulative proportion of no-CRPC patients was 64.4% at Year 1 and 43.4% at Year 2 (Figure 1A). The median survival time until CRPC was 33.3 months (95% CI 23.2–43.4) in patients with a Gleason score of 8 or less. Comparatively, the median survival time until CRPC was 15.7 months (95% CI 13.0–18.4) in patients with a Gleason score of 9 or more. The CRPC-free survival rates during Year 2 were 58.2% with a Gleason score of 8 or less and 33.2% with a Gleason score of 9 or more (*p* < 0.001, Figure 1B).

Based on the multivariate logistic analysis, life expectancy (odds ratio [OR] 0.95, 95% CI 0.91–0.99, *p* = 0.014) and Gleason scores (≥9 vs. ≤8; OR 0.43, 95% CI 0.24–0.75, *p* = 0.003) were associated with cases that did not progress to castration-resistant prostate cancer within two years (Table 2). The multivariate Cox model revealed that life expectancy (hazard ratio [HR] 0.951, 95% CI 0.904–0.999, *p* = 0.0491), BMI (HR 0.870, 95% CI 0.783–0.967, *p* = 0.0101), and CCI (≥2 vs. <2; HR 2.018, 95% CI 1.103–3.693, *p* = 0.0227) were significant predictive factors for OS (Table 3).

## 4. Discussion

Approximately 50% of patients with mHSPC who were treated with ADT did not exhibit progression to CRPC within a two-year timeframe. However, these patients typically had a short life expectancy and presented with a Gleason score of 8 or less. Based on these observations, it was determined that the absence of disease progression to CRPC within two years, along with factors such as short life expectancy, low BMI, and the presence of two or more CCIs, significantly influenced OS.

In terms of OS and effective tumor control, combination therapies utilizing novel agents, either in doublet or triplet regimens, should be prioritized as the first-line standard of care for patients with mHSPC. Nevertheless, the selection of the initial treatment regimen should take into account several other critical factors. First, the balance between quality of life and the tolerability of new pharmacological interventions is essential. For instance, during clinical trials, docetaxel was associated with the onset of febrile neutropenia in approximately 10% of participants [13,14]. Furthermore, elderly patients, who are underrepresented in clinical trials, demonstrated increased toxicity likely due to pre-existing conditions such as anemia and overall fragility, which may affect tolerance to treatments like docetaxel [15]. On the other hand, novel hormonal agents have shown to be relatively more tolerable, exhibiting fewer adverse effects, thus potentially enhancing quality of life when compared to docetaxel over the course of a year [16]. However, the burden of treatment adherence due to the number of tablets required and the extended durations before observing disease progression are additional considerations that must be weighed when selecting the optimal therapeutic approach.

Second, the selection of treatment regimens for mHSPC must carefully consider patient-specific factors such as life expectancy and comorbidity profiles. It is paramount to focus on reducing progression risks and prolonging OS, particularly in younger mHSPC patients who have fewer comorbid conditions. Conversely, patients characterized by a lower life expectancy or significant comorbidities often face a higher risk of mortality unrelated to the progression of mHSPC. Moreover, a lower BMI may correlate with a greater burden of cancer and a diminished quality of life during the course of treatment [17]. The phenomenon known as the ‘obesity paradox’—where individuals with obesity may experience longer survival times—is not yet fully elucidated in the context of mHSPC but might be pertinent to the findings of this study. Adipose tissues, serving as nutrient reserves, potentially provide survival benefits under stress conditions, such as those induced by anticancer treatments that might lead to cachexia [18]. The association between weight loss, particularly muscle wasting, and increased mortality underscores the importance of managing nutrition and promoting exercise training to prevent frailty in elderly patients [19]. This holistic approach to patient care is critical in optimizing treatment outcomes and enhancing the quality of life for those affected by mHSPC.

Third, social factors such as medical costs and insurance coverage must be considered when selecting treatment options. Socioeconomic conditions specific to each patient can significantly influence decisions regarding the sensitivity and suitability of various treatments. For cancer patients, factors such as work-related impairments and high out-of-pocket expenses have been identified as significant contributors to adverse health outcomes [20]. These socioeconomic pressures can complicate the decision-making process, particularly when considering the adoption of relatively expensive novel therapeutic agents that are administered over prolonged periods. The financial burden imposed by these treatment options can be substantial, making it essential to evaluate the economic feasibility for patients when planning personalized cancer treatment strategies.

The classification of a patient as geriatric should be determined not solely based on their chronological age but rather on a comprehensive assessment of their overall health status and anticipated life expectancy. The process of estimating life expectancy for older cancer patients is inherently complex, often complicated by a wide range of variables including diverse comorbidities, functional limitations, and cognitive impairments [21]. Within the context of advanced cancer care, there is a tendency among clinicians to overestimate the life expectancy of their patients [22]. This optimistic bias can inadvertently lead to missed opportunities for timely discussions regarding viable treatment options or necessary preparations for end-of-life care. In the context of our research, life expectancy emerged as a critical predictor of mCRPC progression and OS, underscoring the importance of accurate life expectancy assessments in the management and treatment planning for this patient population.

In our study, a Gleason score of 9 or more, corresponding to grade group 5, emerged as a significant predictor of cancer progression. Notably, grade groups 4 and 5 collectively accounted for 83.0% of the mHSPC cases analyzed, with grade group 5 identified as one of the most severe prognostic factors influencing patients’ survival outcomes [23]. Furthermore, grade group 5 was associated with an increased prevalence of mismatch repair gene mutations, which are characterized by aggressive clinical and pathological features. Despite these challenges, grade group 5 showed a responsive sensitivity to novel hormonal therapies and pembrolizumab, suggesting potential therapeutic avenues [24]. Consequently, our findings suggest that intensified treatment regimens incorporating novel agents may offer more effective outcomes than ADT monotherapy, particularly in cases characterized by high Gleason scores. This underscores the necessity for tailored treatment strategies that consider the unique genetic and pathological profiles of each patient’s cancer.

Transitioning from mHSPC to mCRPC can often lead to a deterioration in health-related quality of life [25]. Therefore, delaying the onset of symptoms associated with CRPC becomes a clinically relevant endpoint in the management of the disease. Skeletal-related symptoms, which are closely linked to pain and overall health-related quality of life, typically manifest approximately one year following the diagnosis of mCRPC [26]. Moreover, these skeletal-related events impose a substantial economic burden on both healthcare systems and patients [27]. Thus, selecting the appropriate systemic treatment for mHSPC becomes paramount as it has the potential to delay the onset of these symptomatic events [28]. By focusing on treatments that effectively delay disease progression and alleviate symptoms, clinicians can ultimately improve the overall quality of life for patients living with advanced prostate cancer.

The survival benefit of intensification treatment had been proved by various clinical trials. Clinicians should change our practice from ADT monotherapy to intensification treatments. However, in the real world, ADT monotherapy still remains the common regimen in treating mHSPC. Receipt of intensification treatment was found to be associated with the presence of specialists and care implementation [29]. Therefore, gradual promotion about the intensified treatment and convenience of access to new treatment methods are necessary. Our study does not advocate against using intensified treatments in mHSPC. In mHSPC, intensification treatment is deemed necessary, but it may not be easily accessible for individuals who have difficulty with oral intake or cannot afford the medication costs due to financial vulnerability. Nevertheless, we should use intensification treatment for patients. This study aimed to underscore the importance of intensification treatments, raising awareness by demonstrating the prognosis of individuals treated with ADT monotherapy. Patients with high Gleason scores who were treated with only ADT monotherapy had a very short median survival time until CRPC of 15.7 months. However, our study has some limitations. First, this study was based on a retrospective analysis with potential selection bias. The treatment was selected by the clinicians, so selection bias is likely to exist. The database did not include any genetic backgrounds. Second, this cohort did not include shifts in the treatment paradigm, such as doublet or triplet therapy. However, it has already been established that intensified treatment performs much better than ADT monotherapy in various aspects. Future studies on ventilation and education about new intensified treatment are necessary, and additional care is needed for the small proportion of patients who do not receive such treatment.

## 5. Conclusions

Despite the evolving landscape of mHSPC treatment, ADT without intensified treatment remains a prevalent approach in clinical practice for de novo mHSPC. Notably, our findings reveal distinct patient profiles associated with differing outcomes. Patients with a prolonged life expectancy and a Gleason score of 9 or higher were observed to have a higher likelihood of transitioning to mCRPC during their lifetime. Conversely, individuals characterized by a shorter life expectancy, lower BMI, and deteriorating comorbidity status were more prone to succumb to mortality before progressing to CRPC. While intensified treatment strategies are deemed indispensable for achieving favorable oncologic outcomes in mHSPC, the principle of shared decision making assumes paramount importance, particularly for patients who may not derive substantial benefit from such interventions. This underscores the necessity for a personalized approach to treatment selection, taking into account individual patient characteristics, preferences, and prognostic factors. Furthermore, our findings highlight the imperative for further prospective studies to deepen our understanding of the complex interplay between patient characteristics, treatment outcomes, and disease progression in mHSPC. By elucidating these relationships through rigorous scientific inquiry, we can refine clinical practice guidelines and optimize therapeutic decision making to enhance patient care and improve overall treatment outcomes.

## Figures and Tables

**Table 1:** Baseline characteristics.

	Total (*n* = 261)	CRPC (*n* = 126)	No-CRPC (*n* = 135)	*p*-Value
Age (yr)	69.2 ± 8.7	67.8 ± 10.0	70.5 ± 7.3	**0.015**
Life expectancy (yr)	17.0 ± 20.6	19.4 ± 29.0	14.7 ± 5.3	0.080
BMI (kg/m^2^)	23.4 ± 3.1	23.6 ± 3.2	23.3 ± 3.1	0.430
ECOG-PS				0.332
<2	230 (88.1%)	108 (85.7%)	122 (90.4%)	
≥2	31 (11.9%)	18 (14.3%)	13 (9.6%)	
Charlson comorbidity index				0.951
<2	214 (82.0%)	104 (82.5%)	110 (81.5%)	
≥2	47 (18.0%)	22 (17.5%)	25 (18.5%)	
Initial PSA value	882.1 ± 6246.2	507.5 ± 1064.1	1231.6 ± 8624.9	0.335
Clinical Gleason score				**0.001**
≤8	106 (40.6%)	38 (30.2%)	68 (50.4%)	
≥9	155 (59.4%)	88 (69.8%)	67 (49.6%)	
Visceral metastasis	27 (10.3%)	13 (10.3%)	14 (10.4%)	1.000

CRPC, castration-resistant prostate cancer; BMI, body mass index; ECOG-PS, Eastern Cooperative Oncology Group Performance Status; PSA, prostate-specific antigen; bold letters indicate statistical significance (*p* < 0.05).

**Table 2:** Univariate and multivariate logistic regression to predict non-progression to castration-resistant prostate cancer within two years.

	Total mHSPC Cohort							
	Univariate				Multivariate			
	OR	95%	CI	*p*	OR	95%	CI	*p*
Life expectancy (continuous)	0.95	0.91	0.99	**0.008**	0.95	0.91	0.99	**0.014**
BMI (continuous)	0.97	0.89	1.05	0.429	1.00	0.91	1.09	0.946
ECOG-PS (≥2 vs. <2)	0.64	0.29	1.36	0.248	0.64	0.28	1.42	0.278
Comorbidity (≥2 vs. <2)	1.07	0.57	2.03	0.824	0.93	0.47	1.83	0.840
Initial PSA (continuous)	1.00	1.00	1.00	0.514	1.00	1.00	1.00	0.627
Clinical Gleason score (≥9 vs. ≤8)	0.43	0.25	0.70	**<0.001**	0.43	0.24	0.75	**0.003**
Visceral metastasis (yes vs. no)	1.01	0.45	2.26	0.989	0.91	0.38	2.23	0.835

mHSPC, metastatic hormone-sensitive prostate cancer; OR, odds ratio; CI, confidence interval; BMI, body mass index; ECOG-PS, Eastern Cooperative Oncology Group Performance Status; PSA, prostate-specific antigen; bold letters indicate statistical significance (*p* < 0.05).

**Table 3:** Univariate and multivariate analysis of factors affecting overall survival in the non-castration-resistant prostate cancer cohort.

	No-CRPC Cohort							
	Univariate				Multivariate			
	HR	95%	CI	*p*	HR	95%	CI	*p*
Life expectancy (continuous)	0.946	0.898	0.997	**0.0373**	0.951	0.904	0.999	**0.0491**
BMI (continuous)	0.885	0.803	0.975	**0.0137**	0.870	0.783	0.967	**0.0101**
ECOG-PS (≥2 vs. <2)	0.820	0.296	2.277	0.7040	0.528	0.177	1.574	0.2518
Comorbidity (≥2 vs. <2)	2.294	1.281	4.108	**0.0052**	2.018	1.103	3.693	**0.0227**
Initial PSA (continuous)	1.000	1.000	1.000	0.4639	1.000	1.000	1.000	0.2924
Clinical Gleason score (≥9 vs. ≤8)	1.455	0.866	2.446	0.1566	1.724	0.957	3.106	0.0698
Visceral metastasis (yes vs. no)	1.294	0.610	2.746	0.5024	1.662	0.722	3.824	0.2323

CRPC, castration-resistant prostate cancer; HR, hazard ratio; CI, confidence interval; BMI, body mass index; ECOG-PS, Eastern Cooperative Oncology Group Performance Status; PSA, prostate-specific antigen; bold letters indicate statistical significance (*p* < 0.05).

## Data Availability

The datasets generated and/or analyzed during the current study are not publicly available due to privacy/ethical restrictions but are available from the corresponding author upon reasonable request.
